# Immunization with a Live Attenuated H7N9 Influenza Vaccine Protects Mice against Lethal Challenge

**DOI:** 10.1371/journal.pone.0123659

**Published:** 2015-04-17

**Authors:** Xiaolan Yang, Jianyu Zhao, Cheng Wang, Yueqiang Duan, Zhongpeng Zhao, Rui Chen, Liangyan Zhang, Li Xing, Chengcai Lai, Shaogeng Zhang, Xiliang Wang, Penghui Yang

**Affiliations:** 1 Beijing Institute of Microbiology and Epidemiology, State Key Laboratory of Pathogen and Biosecurity, Beijing, China; 2 China Astronaut Research and Training Center, Beijing, China; 3 Beijing 307 Hospital Affiliated to Academy of Medical Sciences, Beijing, China; 4 Beijing 302 Hospital, Beijing, China; Thomas Jefferson University, UNITED STATES

## Abstract

The emergence of severe cases of human influenza A (H7N9) viral infection in China in the spring of 2003 resulted in a global effort to rapidly develop an effective candidate vaccine. In this study, a cold-adapted (ca), live attenuated monovalent reassortant influenza H7N9 virus (Ah01/AA ca) was generated using reverse genetics that contained hemagglutinin (HA) and neuraminidase (NA) genes from a 2013 pandemic A H7N9 isolate, A/Anhui/01/2013 virus (Ah01/H7N9); the remaining six backbone genes derived from the cold-adapted influenza H2N2 A/Ann Arbor/6/60 virus (AA virus). Ah01/AA ca virus exhibited temperature sensitivity (ts), ca, and attenuation (att) phenotypes. Intranasal immunization of female BALB/c mice with Ah01/AA ca twice at a 2-week interval induced robust humoral, mucosal, and cell-mediated immune responses in a dose-dependent manner. Furthermore, the candidate Ah01/AA ca virus was immunogenic and offered partial or complete protection of mice against a lethal challenge by the live 2013 influenza A H7N9 (A/Anhui/01/2013). Protection was demonstrated by the inhibition of viral replication and the attenuation of histopathological changes in the challenged mouse lung. Taken together, these data support the further evaluation of this Ah01/AA ca candidate vaccine in primates.

## Introduction

Since the first notification at the end of March 2013, China has been reporting cases of human infection with H7N9 virus to the World Health Organization. This outbreak was the first identification of infection with this virus in humans [1–3]. As of June 2014, a total of 450 laboratory-confirmed cases of human infection with the avian influenza A (H7N9) virus, resulting in 165 deaths, have been identified and reported to the WHO. The Chinese National Health and Family Planning Commission reported 435 of these cases. To date, this virus does not appear to be transmitted easily from person to person, and sustained human-to-human transmission has not been reported [3].

Vaccination is the most effective method of preventing infection by influenza viruses and the severe outcomes thereof. Safe and effective vaccines have been available and used for more than 60 years [4]. Among healthy adults, influenza vaccination can prevent 70–90% of influenza-specific illnesses. Among the elderly, the vaccine reduces the frequencies of severe illness and complications by up to 60%, and deaths by 80% (http://www.who.int/influenza/vaccines/use/en/). Unlike the inactivated influenza vaccine, a live attenuated influenza vaccine (LAIV) is administered intranasally (i.n.), and more closely mimics the natural process of influenza virus infection. Previous researches have revealed that LAIV induces robust humoral, mucosal and cell-mediated immune responses, thus providing strong protective efficacy and long-lasting immunity [5]. In addition, the reduced viral quantities needed for a LAIV yields an increased vaccine production capacity, which results in more rapid supply of a safer influenza vaccine to several developing countries [6, 7].

To date, three cold-adapted influenza A virus strains, A/Ann Arbor/6/60 (AA) (H2N2), A/Leningrad/134/17/57 (H2N2), and A/Leningrad/134/47/57 (H2N2), and two B virus strains, B/Ann Arbor/1/66 ca and B/USSR/60/69 ca have been developed as a LAIV Master Donor virus to generate seasonal influenza vaccines for clinical use in humans [7–9] We reported previously a successful rescue of *ca*, *ts* and *att* reassortant influenza A H1N1 viruses in a short period of time using reverse genetic technology. The cold-adapted, attenuated, and live 2009 pandemic H1N1 vaccines elicit a high level of antibodies response that provide effective protection against wt H1N1 virus infection in animal models, demonstrating the potential of CAIVs [5].

In this study, a live attenuated A H7N9 virus was generated containing the HA and NA genes of A H7N9 virus (A/Anhui/01/2013) isolated at the beginning of the 2013 influenza A H7N9 virus outbreak in China in the background of the A/Ann Arbor/6/60 ca virus using reverse genetics. Phenotypes and protective efficacy of the candidate Ah01/AA ca virus was evaluated *in vitro* and *in vivo*. Thus, our preclinical data support the further evaluation of the Ah01/AA ca vaccine in clinical trials.

## Materials and Methods

### Ethics Statement

Female BALB/c mice, 4–6 weeks old, were purchased from the Laboratory Animal Center of Beijing Institute of Microbiology and Epidemiology and housed under standard temperature, light, and dark cycles. This study was specifically approved by the Institutional Animal Care and Use Committee (IACUC) and ethics committee of Beijing Institute of Microbiology and Epidemiology (ID: SYXK2012-005). All facilities were also accredited by the Animal Care and Ethics Committee of Beijing Institute of Microbiology and Epidemiology. Live-virus experiments were performed in Bio-safety Level 3 facilities in accordance with governmental and institutional guidelines.

Subject provided written informed consent for participation in this study.

### Viruses and cells

The human influenza A H7N9 virus (A/Anhui/01/2013, abbreviated as Ah01/H7N9) used in this study was isolated from a confirmed H7N9-infected patient in the Anhui province in 2013 [10]. The genomic sequences of Ah01/H7N9 are available in the GISAID database under accession numbers EPI509120-EPI509127. Virus stocks were propagated in SPF chicken embryos (Beijing Laboratory Animal Center). The 50% cell culture infectious dose (CCID_50_) for each virus was determined by serial titration of the virus in Madin-Darby canine kidney (MDCK) cells.

### Transfection of cloned cDNA

For each A H7N9 reassortant virus, the six plasmid DNAs encoding the internal protein genes of the AA ca were combined with the two plasmids encoding the HA and NA surface antigen genes from *wt* Ah01/H7N9. Detailed protocols were performed as previously described [5]. The allantoic fluid was harvested from eggs and tested for haemagglutination (HA) activity.

### Phenotypic analysis of the reassortant virus

The *ca* and *ts* phenotypes of the Ah01/AA ca virus were determined as described in detail previously [11, 12].

### Pathogenicity studies in mice

BALB/c mice (n = 5) under sodium pentobarbital anesthesia (60–80 mg/kg) were inoculated i.n. with serial 10-fold dilutions of the Ah01/AA ca virus to determine the pathogenicity. The survival rates of mice were monitored and recorded daily until 14 days. Mice showing 30% of body weight loss were considered to have reached the experimental end point and were humanely euthanized. To evaluate viral replication in various organs, mice were inoculated i.n. with 10^6^ CCID_50_ of either the Ah01/H7N9 or Ah01/AA ca virus. Three days p.i., nasal turbinates, lungs, and brains of infected mice were collected and homogenized in DMEM medium to produce a 10% w/v tissue homogenate. Next, tissue homogenates were obtained by centrifugation and were titrated in MDCK cells. Viral titers of tissue were calculated and expressed as Log CCID_50_/g.

### Immunogenicity and protection from the virus challenge in mice

Groups of mice under sodium pentobarbital anesthesia (60–80 mg/kg) was immunized i.n. twice, in 14 days apart, with different viral doses containing 10^4^ CCID_50_, 10^5^ CCID_50_, or 10^6^ CCID_50_ of each reassortant virus in a total volume of 20 μl, respectively, and sera were collected at 2 weeks after prime and boost. The serum hemagglutination-inhibition (HI) titers were determined by standard methods using 4HA units’ viruses. Serum neutralization antibody titers were tested by micro-neutralization (MN) assays as described previously [13]. Nasal wash and bronchoalveolar lavage fluid (BALF) samples were collected 14 days after boost, and the spleens of vaccinated mice were harvested for assessment of cellular immune responses.

Mice were immunized i.n. with 10^4^ CCID_50_, 10^5^ CCID_50_, or 10^6^ CCID_50_ of Ah01/AA ca virus or PBS (mock-immunized) in a total volume of 20 μl in two doses spaced 2 weeks apart. Mice under sodium pentobarbital anesthesia (60–80 mg/kg) were challenged with the *wt* Ah01/H7N9 virus (50-fold the LD_50_) 14 days after the second immunization. Challenged mice were monitored and recorded daily in terms of body weight and survival for 14 days. In this experiment, no animals exhibit any adverse health or behavior symptoms without intervention. Notably, 10% animals in control group that died in the survival study reached the humane endpoint. Mice showing > 30% of body weight loss was considered to have reached the experimental end point were humanely euthanized by cervical dislocation and then carefully observed and palpated to ensure that there was no respiration and heartbeat. Lung tissues were collected, homogenized, and titered using MDCK cells at 3 days post-challenge. Viral titers were calculated using the Reed and Muench method and are expressed as log_10_ CCID_50_/g lung tissue. Histopathological changes in the lung were assessed at 3dpi by H&E staining and subsequent visualization under a microscope.

### Measurement of mucosal IgA antibody levels by indirect ELISA

The levels of mucosal IgA antibodies against influenza A (H7N9) antigen were determined separately by indirect enzyme-linked immunosorbent assays. Notably, 96-well ELISA plates were coated with 400 ng of purified H7N9 viral antigens. Detailed protocols were performed as previously described [13]. This experiment was performed in triplicate to control the accuracy.

### Statistics

All statistical analyses were performed using the GraphPad Prism software. All data are expressed as means ± SD unless otherwise stated. Following an ANOVA, a multiple comparison test was also performed using the GraphPad Prism ver. 5.0 software.

## Results

### Generation of the Ah01/AA ca transfected virus

**Fig 1:**
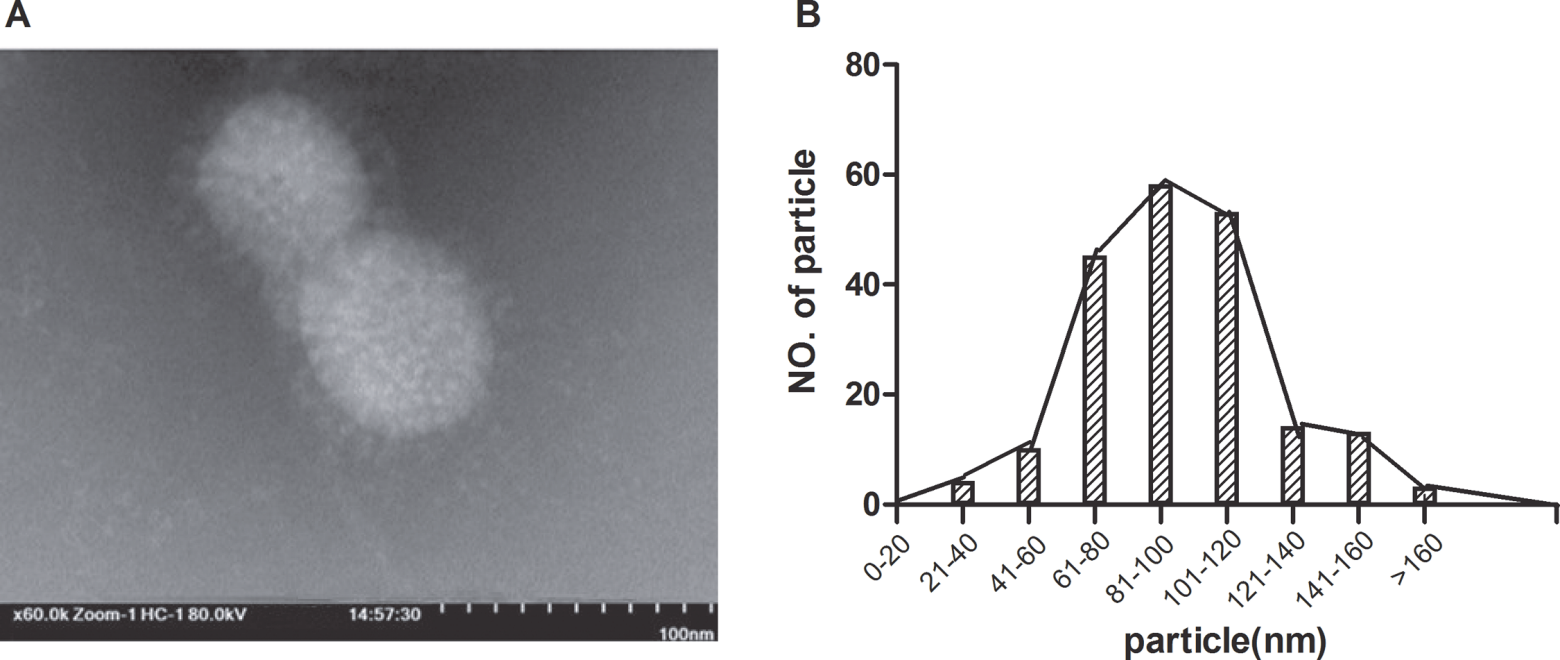
Production of an influenza Ah01/AA ca vaccine candidate using reverse genetics. The characteristics of Ah01/AA ca transfectant viruses were confirmed by visualizing the shape and size distribution of the virus particles. (A) Recombinant Ah01/AA ca virus particles were visualized using electron microscopy. (B) Ah01/AA ca virus particles were measured; 82% (out of 200 particles measured) ranged in size between 80 and 120 nm.

The reassortant Ah01/AA ca viruses were produced through the use of reverse genetics. The infectious titers were 7.5–8.5 log_10_ CCID_50_/ml for the Ah01/AA ca. The reassortant Ah01/AA ca viral particles were examined by electron microscopy and exhibited spherical structures 80–120 nm in size and a typical lipid membrane bilayer on their outer surface (Fig 1A and 1B). Meanwhile, the HA and NA from Ah01/AA ca vaccine was fully sequenced and was identical as that of wt Ah01/H7N9 virus.

### In vitro phenotypes

We and others’ previous reports showed that reassortant influenza H1N1, H3N2, H5N1, H6N1 and H9N2 viruses which six internal gene from the cold-adapted (ca) A/Ann Arbor/6/60 (H2N2) virus exhibit the *ca*, *ts* and *att* phenotypes [14–16]. The Ah01/AA ca viruses replicated efficiently at 25°C and 33°C, but its replication was restricted at 39°C. On the contrary, the *wt* H7N9 parent virus Ah01/H7N9 grew well at both 33°C and 39°C, but its replication was restricted at 25°C (Table 1). These data indicate the *ca* and *ts* phenotypes of the engineered Ah01/AA ca viruses.

**Table 1:** The reassortant influenza H7N9 ca virus is *ca* and *ts* in CEF cells.

Virus	Mean titer±SE (log_10_ CCID_50_/ml)			*ca* ^a^ phenotype	*ts* ^*b*^ phenotype
	25°C	33°C	39°C		
Ah01/H7N9	2.8±0.01	7.0±0.08	6.8±0.03	-	-
Ah01/AA ca	5.9±0.02	6.8±0.06	2.5±0.01	+	+

^a^ *ca* = difference between the mean CCID_50_ at 33°C and 25°C ≥100-fold.

^*b*^ *ts* = difference between the mean CCID_50_ at 33°C and 39°C ≥100-fold.

### In vivo attenuation in mice

As shown in Table 2, the infectious titers of the *wt* Ah01/H7N9 virus in both the nasal turbinates and lungs of infected mice were highly elevated (10^4.6^ and 10^5.2^ CCID_50_/g tissue, respectively). In contrast, Ah01/AA ca replication was restricted to the upper respiratory system (10^1.8^ and 10^1.1^ CCID_50_/g tissue, respectively). Furthermore, no reassortant Ah01/AA ca virus replication was detected in spleen, kidney, and brain at 3 days post inoculation. In addition, inoculation of mice with the reassortant viruses did not result in death, even at the highest dose used (data not shown here).

**Table 2:** Replication of wt and *ca* influenza H7N9 viruses in mice.^a^

Virus	Mean virus titer (log_10_ CCID_50_/g ±SD) on day 3 post inoculation		
	Nasal turbinate^b^	Lung^b^	Brain
Ah01/H7N9	4.6±0.01 ^c^	5.2±0.05 ^c^	1.0±0.02
Ah01/AA ca	1.8±0.10	1.1±0.10	≤1.0 ^d^

^a^ BALB/c mice were inoculated i.n. with 10^6^ CCID_50_ of the Ah01/H7N9 or Ah01/AA ca virus or phosphate-buffered saline (control). These animals were euthanized 3 days after inoculation. Virus titers in nasal turbinate, lung, and brain tissue were determined in MDCK cells. The geometric mean titer ± SD is indicated.

^b^ Significant differences in virus titer between *wt* Ah01/H7N9 virus and Ah01/AA ca virus in the nasal turbinate and lung of mice.

^c^ p<0.02 by Student’s t-test on log-transformed titers.

^d^ Lower limit of detection is 10 CCID_50_/g tissue.

### Ah01/AA ca vaccine induced high antibody responses in mice

**Fig 2:**
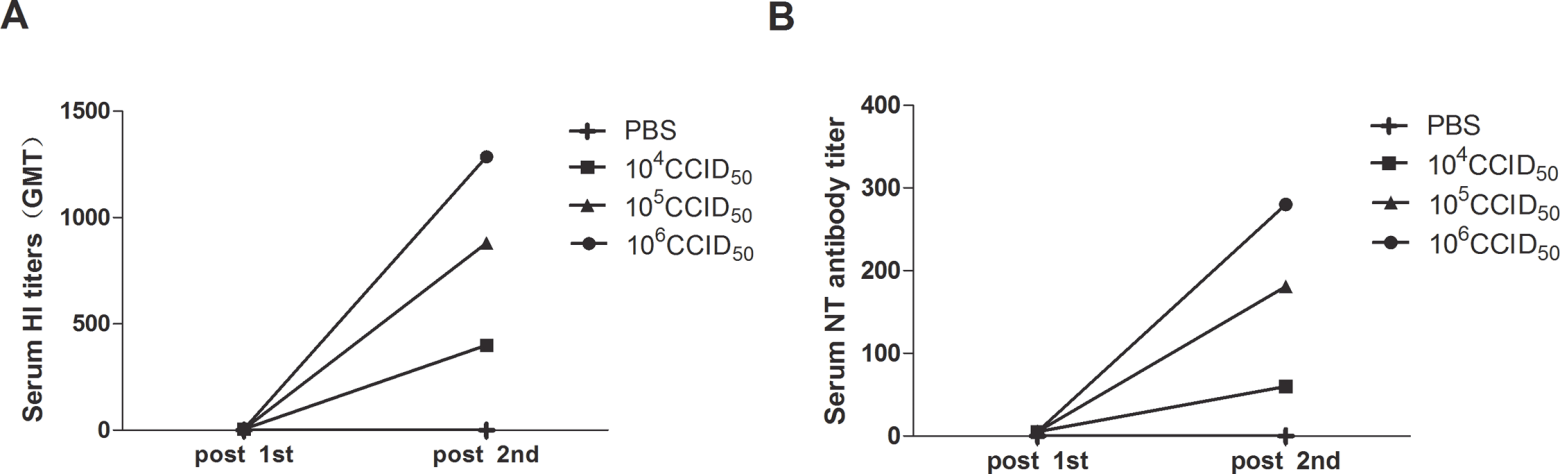
Live attenuated monovalent influenza A (H7N9) vaccines induce antibody responses in mice. Groups of mice were i.n. immunized at weeks 0 and 2 with various doses of 10^4^ CCID_50_, 10^5^ CCID_50_, and 10^6^ CCID_50_ of the Ah01/AA ca vaccine or mock-infected with PBS. Levels of HI (A) and NT (B) antibodies against wt Influenza H7N9 virus in sera 2 weeks after prime and boost. Error bars indicate SDs (n = 8).

Humoral immune responses induced by i.n. immunization of Ah01/AA ca vaccines were assessed by assaying HI and NT antibodies in the serum of vaccinated mice. As shown in Fig 2, the Ah01/AA ca vaccine induced a significantly stronger antibody response on day 14 following the boost in all groups, most notably in the 10^6^ CCID_50_ group. A significant difference in HI titers was observed between the Ah01/AA ca vaccinated group and the PBS control group. Additionally, 14 days following the second vaccination, the HI antibody titer increased sharply to 1,280 in the 10^6^ CCID_50_ group. The serum neutralizing antibody levels were also measured in the mice. Serum NT titers against wt Ah01/H7N9 virus were 60, 180, and 280 in the 10^4^ CCID_50_, 10^5^ CCID_50_, and 10^6^ CCID_50_ vaccine groups, respectively, following two doses of vaccine. Concomitantly, the increase in NT titers in mouse sera corresponds with the change in HI titers.

### Determination of mucosal sIgA antibody levels by ELISA

**Fig 3:**
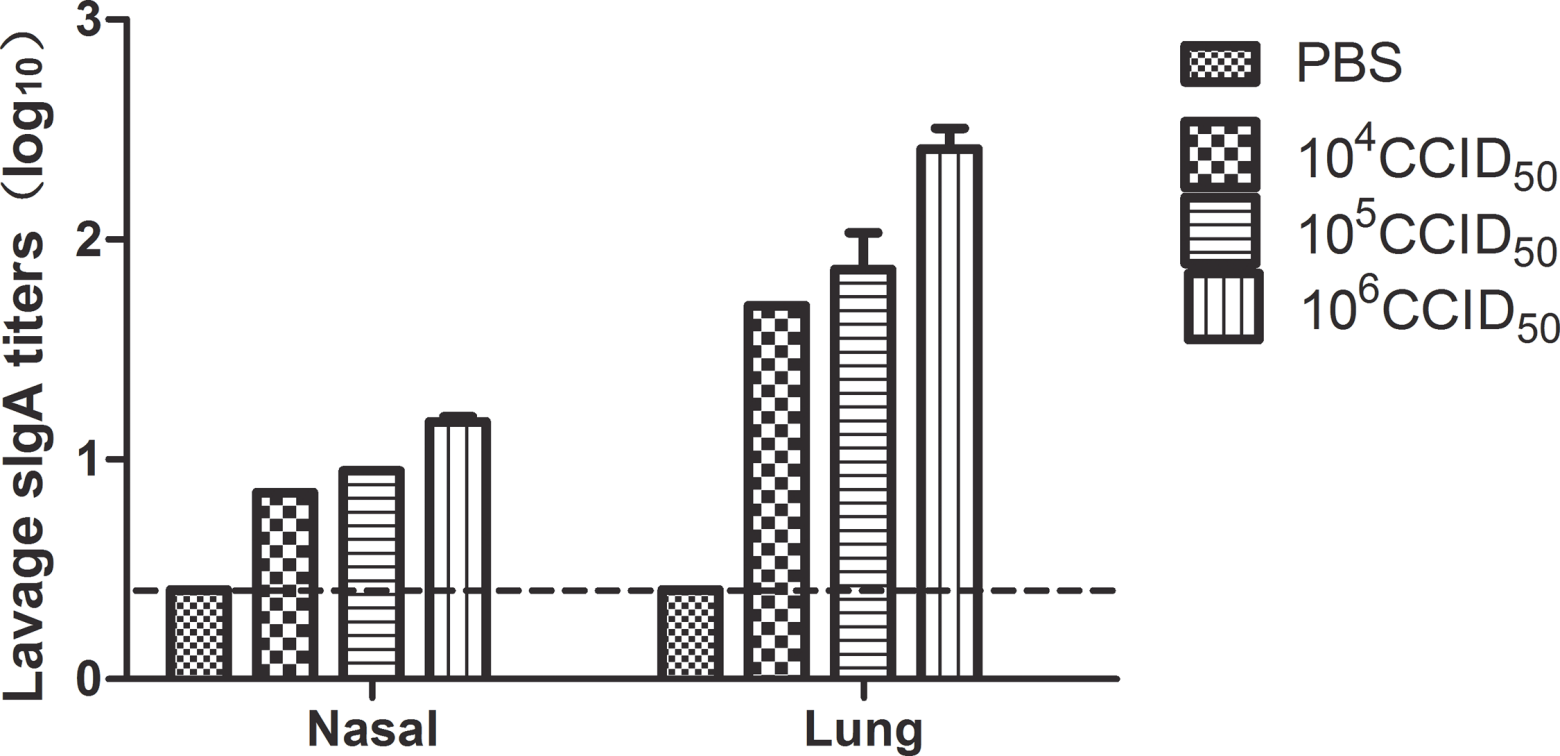
Mucosal antibody response in BALB/c mice. Secretory IgA antibody levels against the influenza Ah01/AA ca vaccine antigen were assessed by ELISA in both nasal and lung lavage fluid from mice i.n. immunized with 10^4^ CCID_50_, 10^5^ CCID_50_, or 10^6^ CCID_50_ of the Ah01/AA ca vaccine or mock-infected with PBS. Nasal and lung lavages were collected 2 weeks after the boost. The values are means ± SD from five mice. * *p* < 0.01 and ** *p* < 0.001.

ELISAs were conducted to determine whether A H7N9 IgA antibodies are detectable in mucosal lavage from mice immunized with the Ah01/AA ca vaccine. In all vaccinated groups, sIgA antibodies against the Ah01/H7N9 virus were tested in the nasal and lung lavages of mice using ELISA plates coated with purified H7N9 viral antigens at 14 days after boost; levels increased in a dose-dependent manner. A viral dose of 10^6^ CCID_50_ Ah01/AA ca elicited a stronger sIgA antibody response in the lung lavage fluid compared to that in the nasal lavage fluid in response to the Ah01/H7N9 virus (titers of 1:290 and 1:16, respectively) (Fig 3). These data suggest that intranasal administration enhances mucosal antibody production in response to A H7N9 vaccination.

### The Ah01/AA ca vaccine induced effective murine cellular immune responses

**Fig 4:**
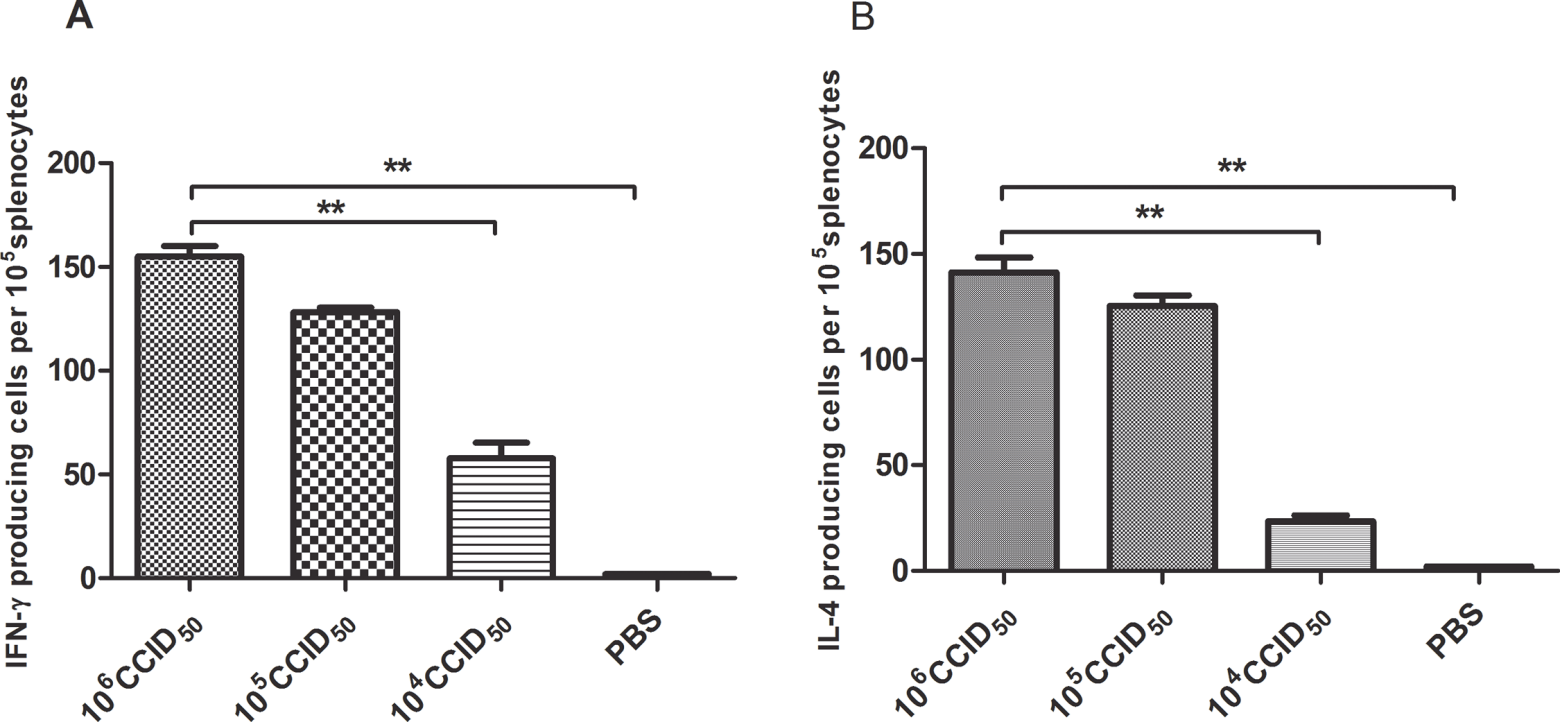
Analysis of IFN-γ and IL-4 by ELISPOT assays. On day 14 following the second immunization, mice were sacrificed and single-cell suspensions were prepared from the spleen, cultured for 48 h, and stimulated with 5 μg/mL purified Ah01/AA viral antigen. IFN-γ (A) and IL-4 (B) secretion by splenocytes was determined by ELISPOT in triplicate wells. Values and bars represent means ± SD. ** *p*<0.001 compared to the PBS group.

To determine the capacity of the Ah01/AA ca vaccine to elicit a cellular immune response, IFN-γ and IL-4–producing cells were enumerated using ELISPOT assays. Splenocytes were harvested at 14 days post boost and stimulated with purified H7N9 viral antigens *in vitro*. As shown in Fig 4A, Ah01/AA ca vaccination led to significantly higher levels of IFN-γ–producing T cells than compared the PBS-treated group; moreover, the effect was dose-dependent (*p*<0.001). Of particular note, no significant difference in the number of cytokine-producing T cells was observed between the 10^6^ CCID_50_ and 10^5^ CCID_50_ vaccinated groups. Additionally, treatment with the Ah01/AA ca vaccine induced significantly more IL-4–producing T cells, in a dose-dependent manner (*p*<0.001), compared to the control group (Fig 4B), suggesting that the Ah01/AA ca vaccine elicited both Th1 and Th2 immune responses. Thus, it would be interesting to measure the ratio of the Th1/Th2 response to assess the type of humoral response that leads to protection.

### The Ah01/AA ca vaccine provides protection from a lethal challenge with live influenza H7N9 virus in mice

**Fig 5:**
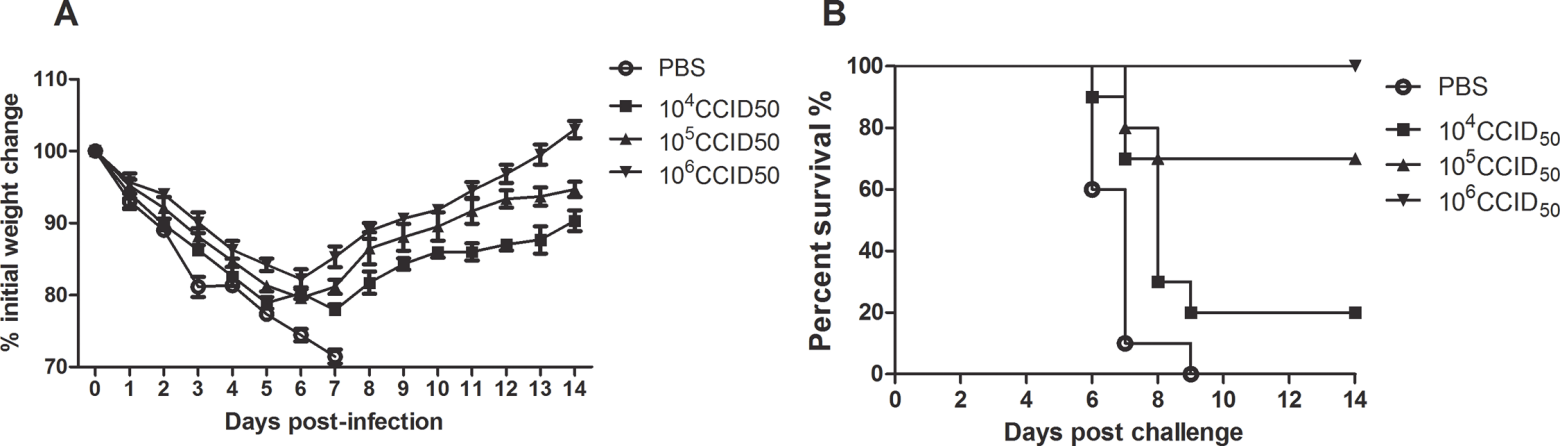
Protective efficacy of Ah01/AA ca vaccine immunized mice against a lethal challenge with live influenza Ah01/H7N9 virus. Two weeks after boost, vaccinated mice were i.n infected with Ah01/H7N9 (50LD_50_) and monitored daily for 2 weeks post challenge. (A) Body weight change (%) of the mice. (B) Survival rate (%). Points represent means ± SD.

To further evaluate the protective efficacy of the Ah01/AA ca vaccine against live H7N9 influenza virus, mice were challenged with wild-type Ah01/H7N9 (50LD_50_) at 2 weeks after boost. In the 10^6^ CCID_50_ vaccinated group 100% mice survived for at least 14 days following challenge with the Ah01/H7N9 influenza virus, and their body weight recovered rapidly at 6 days post infection (Fig 5). On the contrary, mice in the 10^5^ CCID_50_ vaccinated group exhibited a rapid decrease in body weight at day 5 post challenge, which resulted in a 70% survival rate. In contrast, in the 10^4^ CCID_50_ vaccinated group 20% mice survived post challenge. As a comparison, all mice in control group died within 10 days. These results indicate that immunization with 10^6^ CCID_50_ of the Ah01/AA ca vaccine completely protected mice against a live influenza H7N9 viral challenge.

### The Ah01/AA ca vaccine restricts viral replication and attenuates H7N9-induced lung pathology

**Fig 6:**
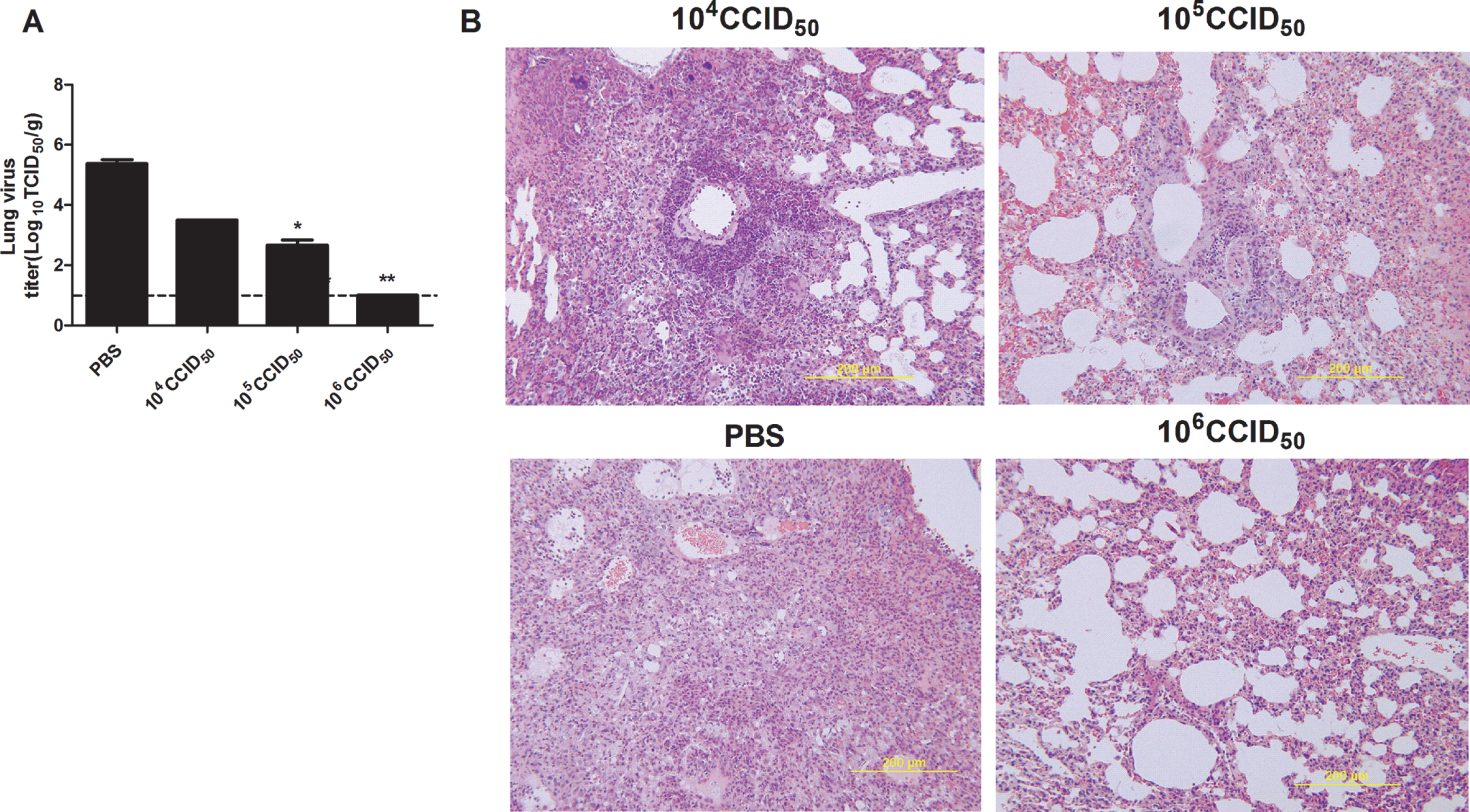
Lung viral titers and histopathological changes of vaccinated mice following challenge with live influenza Ah01/H7N9 virus. Two weeks after the boost, vaccinated mice were i.n. infected with Ah01/H7N9 (50LD_50_) and lung tissues were collected 3 days later. (A) Viral titers in the lung of infected mice. The data were determined in triplicate by CCID_50_ assay in MDCK cells 3 days post infection and expressed as log_10_ CCID_50_/g tissue. Data are means ± SD. *p<0.01; ** *p*<0.001 (B) Lung histopathological changes following virus challenge. Representative histopathological images of lung damage by H&E staining from five mice per group.

To quantify viral replication, lung tissues of infected mice were collected and used to inoculate cultured cells (Fig 6A). Importantly, viral titers in the lungs of mice vaccinated with the Ah01/AA ca vaccine were lower than those in control mice injected with PBS. Interestingly, no virus was detected in the lung tissues of mice immunized with 10^6^ CCID_50_. These results suggest that the Ah01/AA ca vaccine elicited a protective immune response against live influenza H7N9 virus infection.

For histopathological examinations, lung tissues were collected on day 3 post-challenge with live Ah01/H7N9 virus (Fig 6B). Severe histopathological damage, including fragmentation of alveolar walls and infiltration of lymphocytes, was observed in control group, whereas only mild inflammatory changes were observed in mice immunized with Ah01/AA ca vaccine, especially for 10^6^ CCID_50_ group. These data revealed that the Ah01/AA ca vaccine attenuated the severe inflammatory lung pathology caused by infection with the influenza H7N9 virus.

## Discussion

In this present study, we report successful generation of reassortant influenza H7N9 vaccines utilizing the backbone of the A/AA//60 ca donor strain by means of reverse genetics. The Ah01/AA ca viral vaccine exhibits the *ca*, *ts* and *att* phenotypes and is antigenically similar to the wt Ah01/H7N9 parent virus. Two intranasal Ah01/AA ca immunizations, a live virus vaccine, stimulated immune responses and protected mice against a subsequent challenge with wt H7N9 virus in a dose-dependent manner. Different from the previous report[17], the H7N9 ca vaccine virus contained multiple variants with amino acid substitutions in the HA domain could improve the viral growth in eggs. However, these sites mutation impact on the phenotypes and protective efficacy of the reassortant vaccine was not completely clarified. In addition, studies from several groups have already demonstrated that candidate vaccine formulations for the recent H7N9 (2013) can elicit broadly neutralizing not only against homologous virus challenge, but also against heterologous H7 viruses [18]. Of note, this broad-spectrum protection against divergent H7 viruses has been shown for both human and mouse sera, which suggest that perhaps even divergent H7 HAs share a large amount of common epitopes [19]. Specifically, an H7 LAIV on an Ann Arbor (H2) backbone has already been shown to elicit a protective response against a homologous and heterologous challenge [20]. In lines with these reports, we find the LAIV described here can be shown to also elicit a similar broad immune response in this set of experiments. The idea of generating a live attenuated influenza vaccine against H7 was demonstrated by Subbarao and colleagues [20, 21]. However, it would be beneficial if a live attenuated influenza vaccine based on the novel H7N9 that emerged in 2013 could elicit a similar response against other divergent H7 viruses. Overall, our findings demonstrate that reverse genetic engineering methods can be used to insert H7N9 HA and NA genes into a A/AA/6/60 ca donor backbone, which can be used to produce a functional vaccine. These results warrant further testing of the Ah01/AA ca virus as a potential candidate vaccine in human clinical trials. Additional research assessing the immunogenicity and protective efficacy of the Ah01/AA ca virus in ferret, monkey, and human models would also be beneficial.

Vaccines against pandemics are evaluated for safety and immunogenicity in clinical trials, but efficacy data can come only from studies in experimentally infected animals, such as mice, ferrets and monkeys. In this study, we determined that two 10^6^ CCID_50_ doses of the Ah01/AA ca viral vaccine were necessary to completely protect mice against a lethal challenge with live H7N9 virus, as demonstrated by the amelioration of body weight reduction and increased survival rate compared to use of two doses of either 10^4^ CCID_50_ or 10^5^ CCID_50_ in challenge experiments. As shown in Fig 6, the Ah01/AA ca vaccine protected mice from pulmonary virus replication in a dose-dependent manner, as evidenced by viral titer measurements and assessment of lung tissue pathology. Overall, our data suggest that two 10^6^ CCID_50_ doses of the Ah01/AA ca viral vaccine may be effective in pre-clinical animal models.

The need for this specific vaccine is based on the fact that H7N9 subtype influenza viruses have been isolated from patients who were solely PCR positive for the 2013 influenza A (H7N9) virus. Influenza H7N9 viruses can infect humans without prior adaptation, suggesting that they likely present a potential pandemic threat [1]. A crucial strategy for pandemic preparedness is to generate a H7N9 candidate vaccine prior to its actual emergence and spread. The clinical relevance of the present findings requires further investigation in other animal models—such as ferrets and/or monkeys—to determine whether the protective efficacy of a live attenuated H7N9 vaccine could be extended based on the present donor strain. Our findings support the evaluation of this vaccine in clinical trials and human use. A suitable antigenic match with the local pandemic strain is critical; indeed, use of such a strain would save the time required to produce a vaccine in the face of an influenza pandemic.
